# Detection of cracks in concrete using near-IR fluorescence imaging

**DOI:** 10.1038/s41598-023-45917-3

**Published:** 2023-11-02

**Authors:** Andreas Karpf, Michael Selig, Aktham Alchaar, Magued Iskander

**Affiliations:** 1 Civil and Urban Engineering Department, New York University, Six Metrotech Center, Brooklyn, NY 11201 USA; 2 Civil and Urban Engineering Department, New York University, Abu Dhabi, United Arab Emirates

**Keywords:** Civil engineering, Optical techniques, Structural materials

## Abstract

Structural health monitoring of civil infrastructure is a crucial component of assuring the serviceability and integrity of the built environment. A primary material used in the construction of civil infrastructure is concrete, a material that is susceptible to cracking due to a variety of causes, such as shrinkage, creep, overloading, and temperature change. Cracking reduces the durability of concrete structures, as it allows deleterious environmental agents to penetrate the surface, causing such damage as corrosion of steel reinforcement and delamination of the concrete itself. Conventional crack detection techniques are limited in scope due to issues relating to pre-planning, accessibility, and the need for close proximity to the test surface. Contactless optical image monitoring techniques offer the opportunity to overcome these limitations and have the potential to detect cracks at a distance. Concrete has been reported to have a near-infrared (Near-IR) fluorescence line at a wavelength of 1140 nm when excited with red light. This work investigates the use of fluorescence imaging for the detection of cracks in cementitious surfaces using shallow angle incidence excitation red light. Light oriented at a shallow angle does not excite interior surfaces of cracks, which appear as darker features in images of fluorescing concrete. Artificial cracks with widths of 0.2–1.5 mm were readily imaged using a near-IR camera at distances of 0.5 and 1.3 m. An additional concrete sample with a 0.08 mm wide crack was produced using a flexure apparatus and was also imaged. It is worth noting that the 0.08 mm crack was detected despite its width being below the 0.1 mm pixel resolution of the camera, with the aid of digital image enhancement algorithms.

## Introduction

Concrete is perhaps the most widely used building material for urban construction, worldwide. However, concrete structures are susceptible to cracking due to several causes, including short-term plastic shrinkage, mechanical strain from excessive loading, creep, and/or temperature changes^1, 2^. Cracking is a major durability concern for concrete as it allows the penetration of moisture, chlorides, deicing salts in urban environments, and sulfates in harsh environments. The intrusion of such agents causes the deterioration of concrete as well as corrodes the underlying steel reinforcement^3–5^. In this context, cracking directly affects the service life of concrete structures, therefore regular monitoring of civil infrastructure is a vital component of asset management. Early detection of flaws can thus help owners anticipate prospective damage, ensure the serviceability and durability of concrete infrastructure, and reduce their lifecycle^6^ costs.

The most widely used approach for crack detection is visual inspection. However, visual inspections are time-consuming and can easily miss small cracks in large structures. In addition, crack growth is highly related to temperature variation within the structure^7^, so cracks may be missed during contractive periods. Visual inspections are often augmented with acoustic surveys as well as spot material and permeability testing. These techniques require special training and typically involve loss of use of the structure while they are being conducted. In a few cases, monitoring solutions involve embedding sensors into the concrete while it is being poured. This approach requires planning beforehand and post-construction accessibility to the instrumentation. Once cracks have been identified, crack meters can be installed on the surface to monitor crack growth. Use of crack meters suffers from several problems. First, sensors act on a localized basis only. Second, accessing gauges is often difficult. Third, gauges can easily be damaged in unprotected areas. Fourth, this approach is further complicated by the need for large numbers of sensors and the associated wiring to monitor large-scale projects, and long-term gauge durability issues^8^. Recent advances in sensor technology, such as fiber optic strain gauges, can serve to reduce the amount of wiring required, but fiber optic sensors are considered low-durability instruments and require skill for installation and interpretation of the results^9, 10^. In addition, strain gauges do not directly detect cracks, instead, they infer cracking from the redistribution of stresses within structures due to cracking. Finally, it is also important to note that many of the conventional techniques, like crack meters and strain gauges, cannot detect old cracks as they measure relative change after their installation.

Non-contact monitoring techniques are being investigated due to their potential to overcome the drawbacks of conventional instruments. The contact-free nature of such instruments cuts down on labor requirements, is less impacted by accessibility issues, and uses considerably fewer sensors than current technology^11^. The following techniques are being employed:

- **Ultra-sonic** approaches to crack detection have been the most extensively employed^12^. The majority generate ultrasonic pulses while in contact with the surface, and employ known transmission properties of concrete, to identify defects via changes in the time of flight of the acoustic wave. Tomographic methods have also been used to combine readings from many sensors and increase resolution. However, these approaches require equipment that is in close proximity to the surface (on the order of mm) and have limited spatial resolution.
- **Ground penetrating radar (GPR)** uses high-frequency radio waves to assess conditions by capturing the attenuation of reflected radar waves^13^. The emitted waves have specific velocities depending on the permittivity of the material through which they pass, and changes in velocity can indicate anomalies, including concrete delamination and corrosion of steel reinforcement^14^. GPR, however, is functional only at a close distance to the surface and can be difficult to use on vertical surfaces or ceilings.
- **Terrestrial Laser Scanning (a technique similar to LiDAR)** generates 3-dimensional images of a scene with millimeter-level resolution using reflections of light from the target^15^. A complication with this approach is the difficulty in identifying fine cracks without having reflectivity data^16^. However, resolutions of 0.2 mm were achieved, provided that surfaces were cleaned to enhance crack visibility^17^.
- **Infrared thermography** identifies delaminated concrete because of its lower thermal conductivity compared to healthy concrete, so damaged surfaces appear hotter than surrounding intact surfaces when the temperature gradient of concrete surfaces is measured using an infrared camera^18^. Optimal test conditions require continuous solar loading and low wind speed; the former is impossible to achieve for outdoor structures. In addition, the optimal time of day for detecting damage depends on the orientation of the surface, with defects in bridge decks optimally seen at noon, while bridge deck soffit defects are better seen at midnight^19^.

Machine Learning has been used in conjunction with visible light images taken from unmanned aerial vehicles (UAVs) to identify cracks. Here, the UAVs provide a mobile platform for recording images from a distance. Machine learning algorithms such as convolutional neural networks were used to identify cracks from visible light images^20, 21^. The use of a UAV platform shows promise for scanning larger areas of roadways and other surfaces. The machine learning algorithms automate the process of identifying cracks. The use of convolutional neural nets, however, requires significant amounts of computational power^21^, and thus may not be well suited for fieldwork. As the field progresses, it is expected that machine learning algorithms may complement a variety of detection techniques.

Fluorescence imaging has the potential for use in non-contact crack detection. Fluorescence occurs when a substance is excited by incident radiation, undergoes relaxation (i.e., the nuclear positions shift to achieve equilibrium distances corresponding to the new excited state), and then spontaneously de-excites by emitting a photon (i.e., the fluorescence) where the emitted photon is of lower energy than the absorbed one^22^. It has been shown that injecting cracks with an epoxy resin that contains a fluorescent dye can enhance the visibility of cracks. Using fluorescence microscopy, one can use a filter to block the excitation light; light emitted by the fluorescing dye from within the cracks is the primary source of light in the resulting images and is used to identify crack patterns^23, 24^.

As opposed to using a dye, it has also been shown that Portland cement itself fluoresces in the near-IR range (near-IR light covers the wavelength range from about 800–2500 nm). Illuminating a cement sample with long wavelength visible light (*λ* = 550–700 nm), results in the cement fluorescing in the near-infrared (*λ*_peak_ ~ 1140 nm); peak fluorescence occurred with an excitation wavelength between 620 and 640 nm^25^. In the case of concrete, the source of the fluorescence is the silicon crystals in the calcium silicate component of cement^25^. There was no impact on the fluorescence wavelength due to factors such as strain conditions, water/cement ratio, and curing period. The advantage of imaging using IR fluorescence is that one can use a filter to record images at the target wavelength and know that visible features are due only to the fluorescing material (i.e., concrete).

Meng et al.^25^ also demonstrated an approach to crack detection where they coated a concrete surface using opaque paint before stressing the sample. In this case, the paint cracked along with the concrete. This exposed the underlying concrete, resulting in only the fractured parts (cracks) fluorescing under the excitation of a laser perpendicular to the specimen. The limitation of this approach, however, is that it requires concrete to be painted prior to cracking, and it cannot differentiate between cracks in the concrete and areas where just the paint has cracked or flaked off. Additionally, the use of a laser for excitation limits one to analyzing small areas at a time.

The objective of this study is to explore the detection of cracks in concrete using the fluorescence of the silica naturally occurring in the cement. The study improves on past work in a number of ways. First, the concrete was tested as-is without it being painted. Second, the use of a laser was eliminated in favor of LED lights, which permitted illuminating a much wider swath of concrete. Third, the use of a shallow angle of illumination ensured that the interior surfaces of cracks received no excitation, thus permitting the detection of cracks by detecting dark shadows within swaths of the fluorescing concrete surface.

## Methods

**Figure 1:**
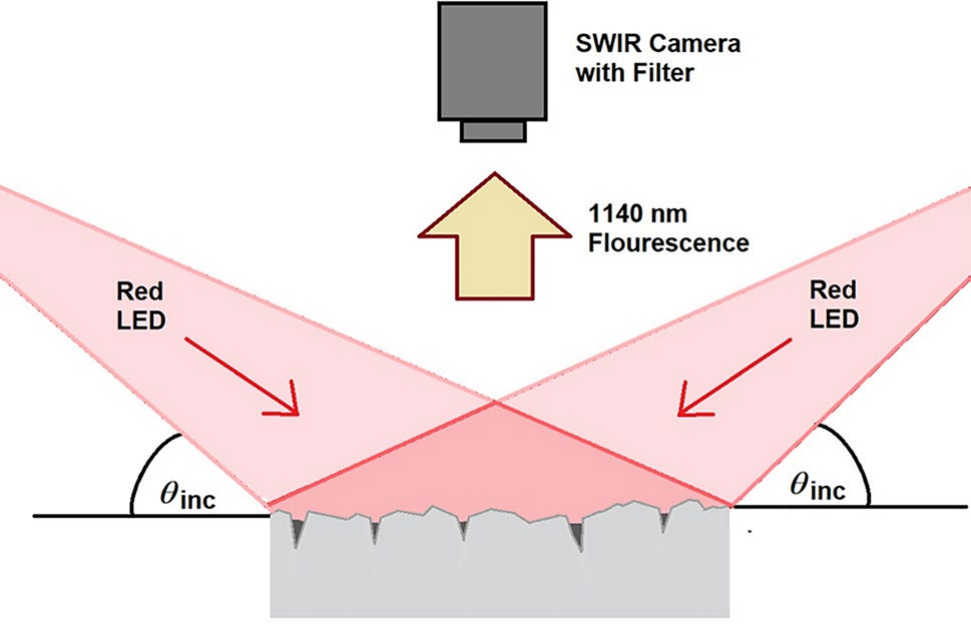
Crack detection using shallow angle excitation of concrete fluorescence. Light from LED bars (*λ*_peak_ = 632 nm) is incident on concrete surfaces from opposing directions at a shallow angle of incidence (θ_*i*_ = 10°–40°). Illumination from both sides with a broad angular range minimized the amount of shaded areas due to irregularities in the surface, and ensured that the only areas not receiving excitation light represented the interior surfaces of cracks.

In this work, a crack-detection approach using fluorescence imaging is demonstrated using the known 1140 nm concrete fluorescence line emitted when excited by light having a wavelength, *λ*, ranging between 550 and 700 nm. Unpainted concrete surfaces were illuminated with red LED light. The most efficient emission of 1140 nm fluorescence occurred when concrete was excited by light having *λ* = 620–640 nm^25^. Red LEDs were selected as the excitation source due to (1) their emission at *λ*_peak_ = 632 nm, which falls within the peak excitation wavelength range, and (2) their broad angle of emission which maximizes the sample area that can be illuminated. Samples were illuminated from opposing sides at a shallow incident angle θ_*i*_ ranging between 10° and 40° (Fig. 1). The shallow angle was used to ensure that the interior surfaces of cracks received no excitation light. As a result, images would show a fluorescing concrete surface with cracks appearing as dark features, due to their lack of fluorescence. Illumination from both sides with a broad angular range minimized the amount of shaded areas due to surface irregularities. Shadows from surface roughness or irregularities would otherwise appear as dark features and complicate crack detection.

A short-wave infrared (SWIR) camera was used to image the fluorescing samples. The camera had a resolution of 640 × 512 pixels and used an *Indium Gallium Arsenide* (InGaAs)-based sensor, which has enhanced sensitivity in the (900–1700 nm range) as opposed to the *Silicon-based* sensors used in typical Visible/Near-IR cameras. This provided a significantly higher signal-to-noise ratio when imaging the 1140 nm fluorescence. The camera was outfitted with a long-pass filter that blocked wavelengths < 1000 nm to permit using it in ambient white LED laboratory lighting, having a spectral range of 400–700 nm. A higher resolution camera is indeed desirable, but the employed camera was among the highest-resolution SWIR cameras available within the required detection range.

**Figure 2:**
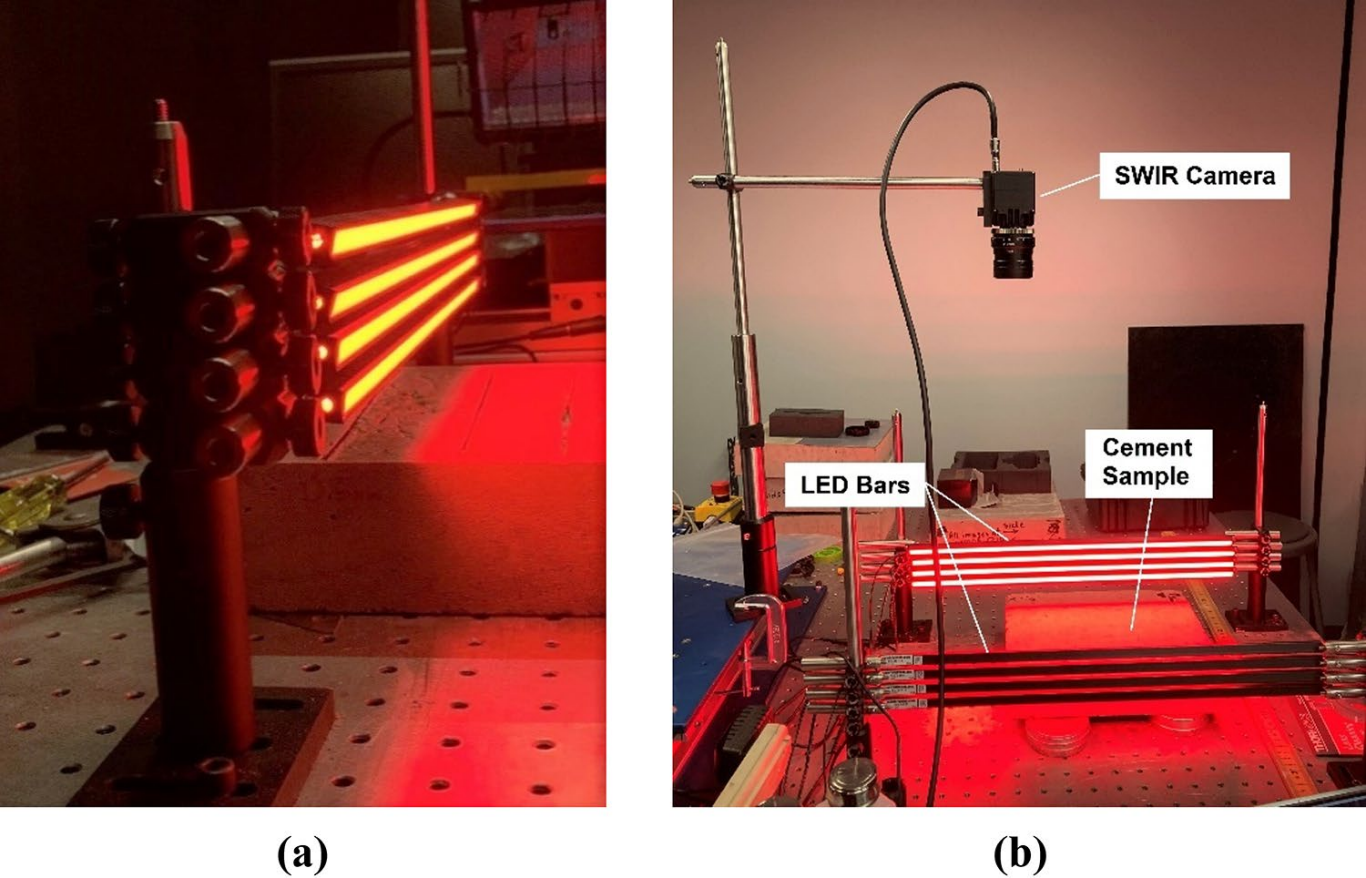
Experimental Apparatus for observing 1140 nm concrete fluorescence. (**a**) Red excitation light (*λ*_peak_ = 632 nm) was provided by four sets of LED bars positioned on either side of the sample. (**b**) Fluorescence was observed using an SWIR camera with long-pass filter positioned directly above the sample.

Samples were illuminated by red LED light bars (45 cm in length); four bars were stacked above and below each other on either side of the sample and oriented so that their axes were parallel to the sample surface (Fig. 2). The LED bars were angled such that their emitted light shined on the samples with an angle of incidence ranging from 10° to 40°. Each LED bar had a luminous flux of 150 Lumens, which resulted in an irradiance of approximately 0.2 mW/cm^2^. This was significantly weaker than the laser light used in the work by Meng et al.^25^ due to the LED light being diffuse and spread over a much larger sample area. Fluorescence was observed by positioning the SWIR camera (with the long pass filter attached to its lens) directly above the sample. To compensate for the resulting weaker fluorescence, images from the SWIR camera were recorded using a 5-s exposure time.

Three concrete samples were fabricated using a concrete mixture having a cement-to-sand-to-aggregate ratio of 1:2:2, by weight. The first sample contained a smooth, unbroken surface; half of the sample was coated with paint, to prevent the observation of fluorescence, while the other half was untreated. This sample was employed to optimize the imaging parameters used to observe concrete fluorescence. The second sample contained artificial cracks of widths 0.2 mm, 0.5 mm, 0.7 mm, 1.2 mm, and 1.5 mm. Artificial cracks were created by inserting plastic sheets of varying thicknesses into the fresh mixture. The sheets were removed after the samples were mostly cured, and the width of the artificial crack thickness was confirmed after the concrete was fully cured. Thickness measurements were carried out using high-resolution images of the cracks and a fiduciary scale. A third concrete sample was created to demonstrate the technique on a “real” crack. This was accomplished by conducting a three-point flexural test on the specimen to introduce real cracks without causing complete fractures. The result was a sample with a single long crack along its center, having an approximate width of 0.08 mm.

## Results and analysis

**Figure 3:**
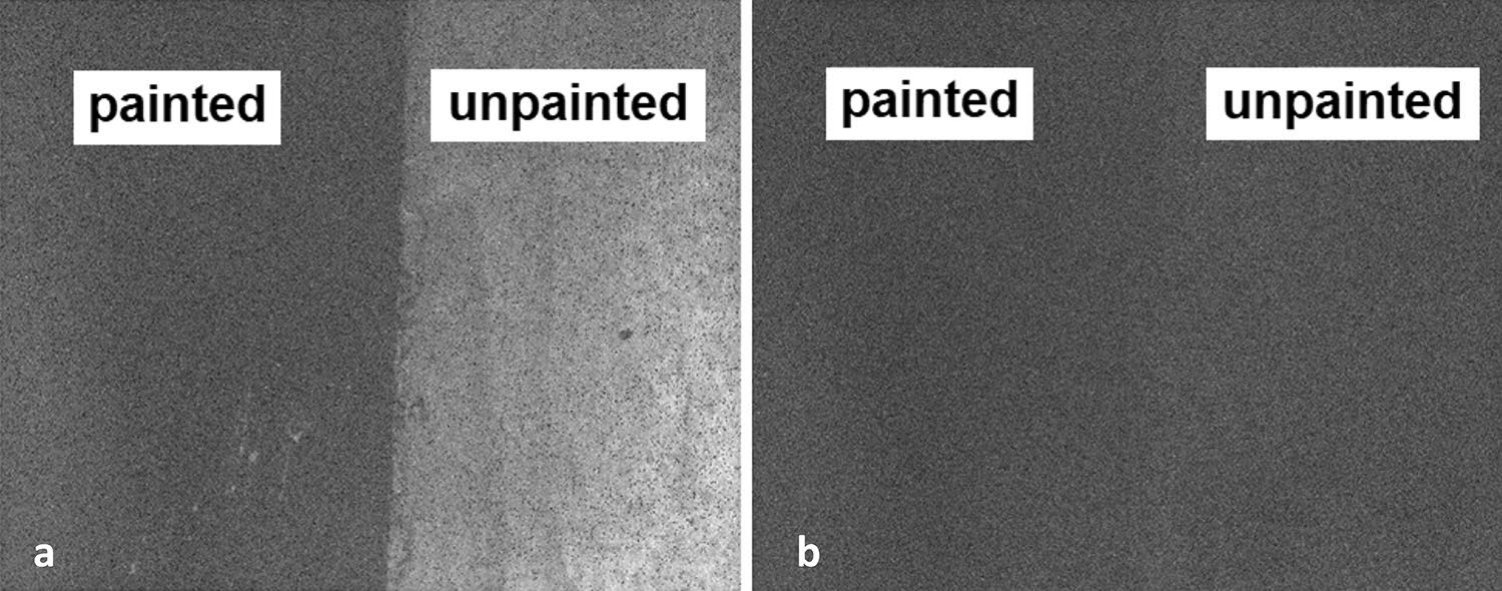
Images of half-painted smooth concrete sample seen through the SWIR camera. (**a**) Sample illuminated using both red excitation LED and ambient room lighting. The right side of the sample was unpainted and is seen to be fluorescing at 1140 nm. (**b**) Sample illuminated using ambient white LED light only.

The observation of concrete fluorescence using the reported approach was first confirmed by inspecting images of the half-painted smooth concrete sample captured using the SWIR camera (Fig. 3). The sample was illuminated with the red LED light; the paint shielded the underlying concrete from the red excitation light, resulting in only the unpainted half exhibiting 1140 nm fluorescence. Figure 3a depicts the sample as seen through the SWIR camera with a combination of ambient white LED room lighting and the red excitation LED turned on. The right side of the sample was unpainted and is seen to be fluorescing at 1140 nm. Figure 3b shows the same sample with the red excitation LED light turned off. On close inspection, one can see evidence of barely detectable fluorescence due to the room’s ambient light containing a small red component^26^. The faint component of red light contained within the room’s white ambient lighting induced low-level fluorescence in the concrete sample. Again, the painted side shows no fluorescence.

**Figure 4:**
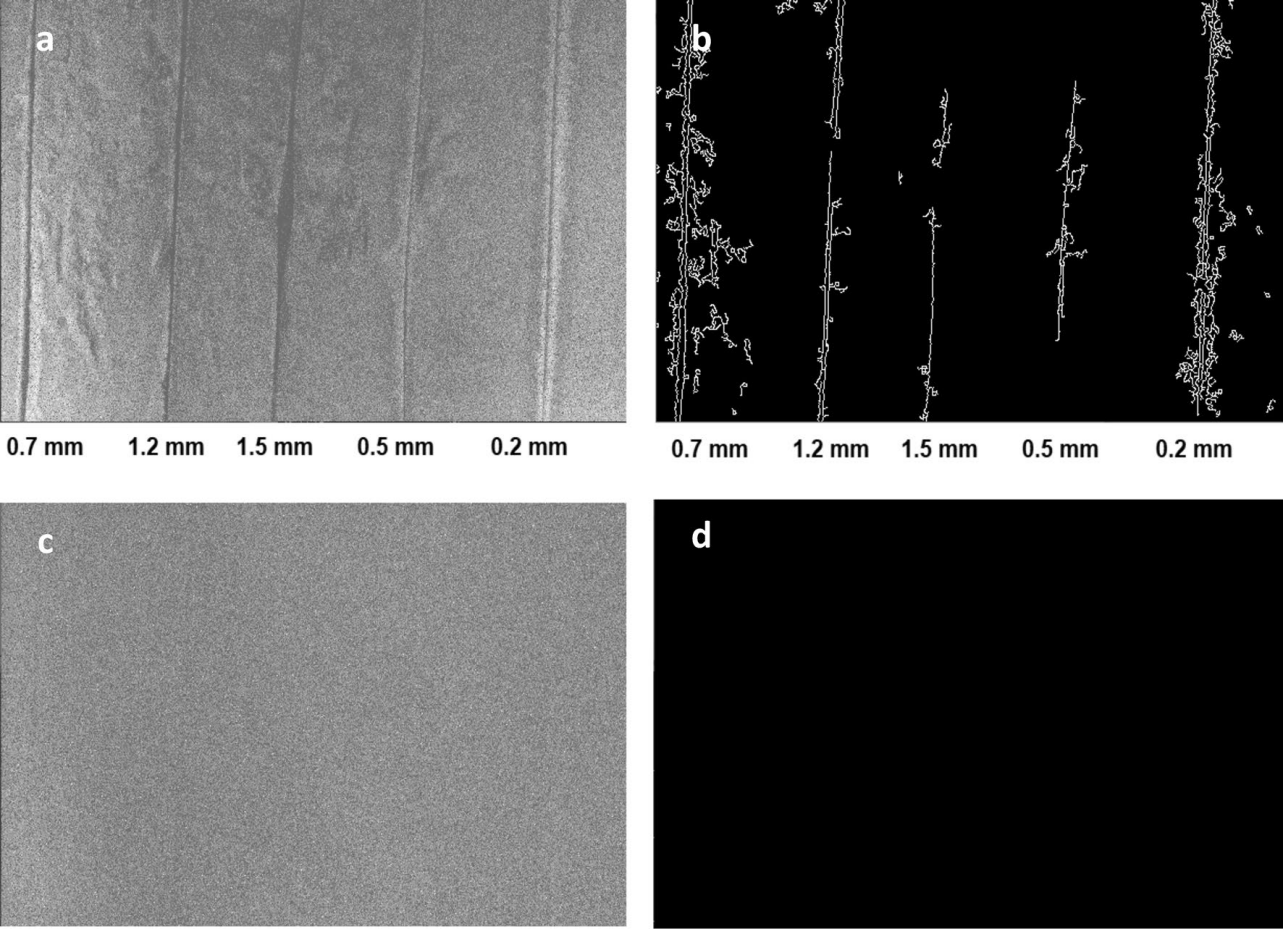
Concrete sample with artificial cracks having widths ranging from 0.2 to 1.5 mm, as imaged through the SWIR camera with the camera located 1.3 m above the sample. (**a**) Averaged image of the sample with the red LED light on. (**b**) Cracks detected after processing using the Canny edge detection algorithm. (**c**) Averaged image with the LED turned off. As expected, no meaningful fluorescence was observed in this control case. (**d**) Image (**c**) after processing using Canny algorithm, where no cracks were detected in the control case.

**Figure 5:**
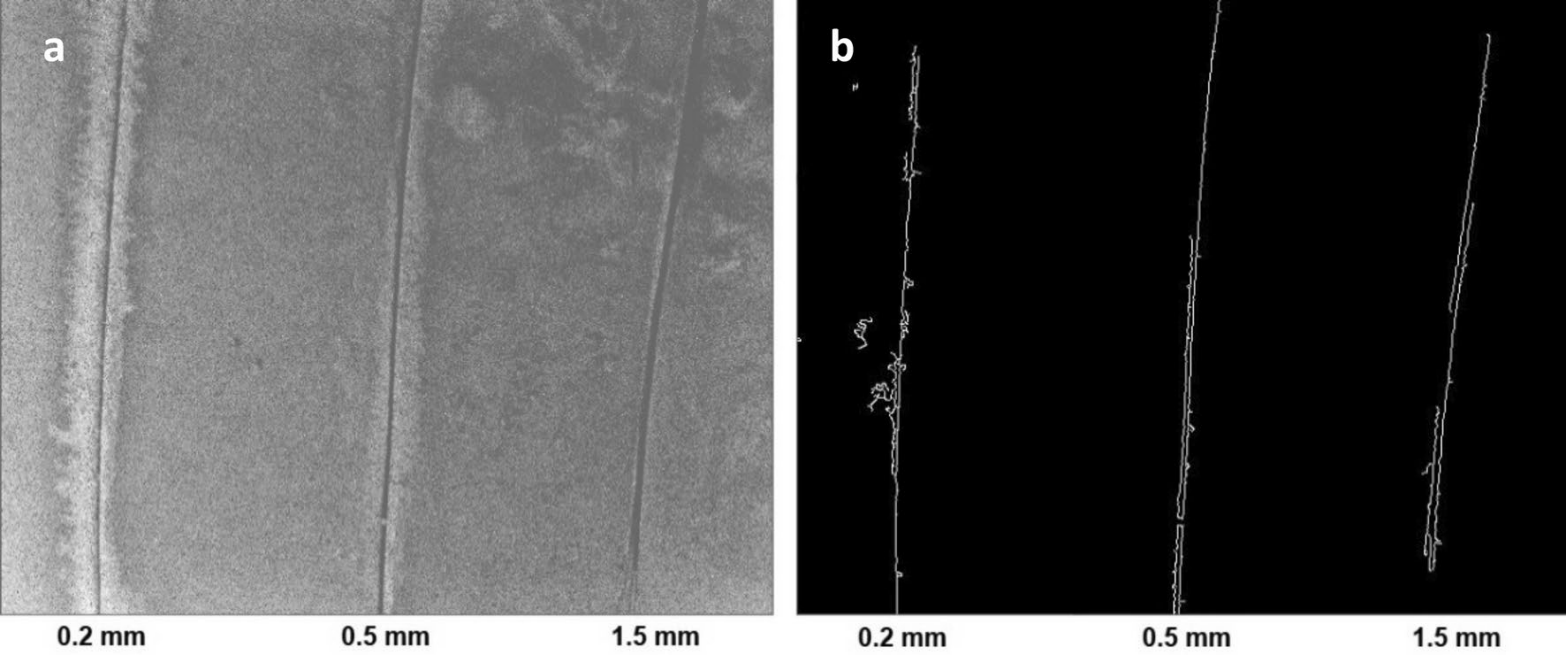
Concrete sample with artificial cracks having widths ranging from 0.2 to 1.5 mm, as imaged through the SWIR camera with the camera located 0.5 m above the sample. (**a**) Averaged image of the sample with the red LED light on. (**b**) Cracks detected after processing using the Canny edge detection algorithm.

To test the capability of the approach for detecting cracks of various sizes, a sample containing artificial cracks of widths 0.2 mm, 0.5 mm, 0.7 mm, 1.2 mm, and 1.5 mm was used. These sizes were selected to balance two concerns. First, sizes were selected to represent a variety of crack widths encountered in practice that are difficult to detect visually. Second, thicknesses were also based on the feasibility of creating artificial cracks of known thickness within the specimen. The apparatus was tested first with the SWIR camera at a distance of 1.3 m from the sample (Fig. 4), corresponding to an image resolution of 0.3 mm per pixel. The test was repeated with the SWIR camera positioned at a closer range of 0.5 m (Fig. 5), corresponding to an image resolution of 0.1 mm/pixel. In both cases, images were recorded with an exposure time of 5 s to maximize the signal-to-noise ratio.

Once captured, the images were processed to enhance the identification of cracks using a routine written in Python. First, ten images were averaged (to minimize the effects of random noise). Next, a thresholding algorithm (THRESH_TRUNC) from the OpenCV library^27^ was used to remove the effect of dead pixels (i.e., a small percentage of pixels in images were completely black—these represented non-responsive or ‘dead’ pixels on the imaging chip). The thresholding function compared each pixel against a set threshold. If the pixel’s value was greater than the threshold, its value was reassigned to the threshold value; if the pixel’s value was less than or equal to the threshold, it retained its original value. A Gaussian filter (using the GAUSSIANBLUR function from OpenCV) was then applied to smooth the averaged image. Lastly, the Canny edge detection algorithm^28^ was used to identify edges (i.e., cracks) in the image. In this routine, gradients for the image are calculated, and an upper and lower threshold are specified to identify edges. Specifically, edges whose gradient is above the upper threshold are automatically treated as edges; those whose gradient is below the lower threshold are deemed non-edges and ignored. Gradients between the upper and lower thresholds are determined to be edges based on their continuity with other potential edge pixels. This testing for the continuity of potential edge pixels in the Canny algorithm helps eliminate false positive signals due to a sample’s surface texture; that is, the disconnected nature of shadows resulting from surface texture are not continuous features and are rejected by the algorithm.

It is worth noting that there were some gaps in the 0.5 mm and 1.5 mm cracks identified using the Canny algorithm shown in Fig. 4b. The gap in the middle region of the 1.5 mm crack corresponds to an area that is considerably wider and shallower than the rest of the crack, resulting in lower contrast between the interior of the crack and the sample surface, thus it was not treated as a crack by the algorithm. The other gap in the 1.5 mm crack, near the top, as well as the gaps in the 0.5 mm crack also appear to have been due to low contrast between the crack and the surrounding cement surface. The post-processing algorithm relied on contrast to identify potential edge pixels. In any case, although the edge detection algorithm included these relatively small gaps, the use of concrete fluorescence at 1140 nm was shown to be capable of identifying cracks whose widths are at or slightly below the camera’s resolution. It is possible that future work combining the results of different post-processing algorithms with different strong points may minimize such gaps in crack identification.

**Figure 6:**
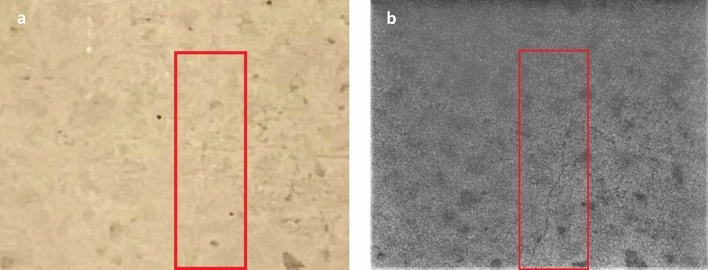
Image of a 0.08 mm wide crack (**a**) imaged using an iPhone camera where the displayed portion of the image has the same resolution as the SWIR camera. (**b**) After being processed using THRESH_TRUNC algorithm. Note that the width of the crack was below the camera’s 0.1 mm resolution.

Extreme environmental conditions such as exposure to seawater and deicing salt require that cracks in concrete not exceed 0.15 mm and 0.18 mm, respectively (the ACI specified damage threshold for concrete in these environments)^29, 30^. Therefore, a concrete sample with a 0.08 mm crack (i.e., a crack that was a factor of two smaller than this threshold) was created using a flexure apparatus. The goal was to demonstrate that the technique could not only detect cracks at this threshold for damage but also identify smaller cracks that could be repaired prior to causing damage. The sample was imaged with the SWIR camera located at a distance of 43 cm from the surface, which was the closest position possible for the available lens. This crack’s width is classified as “Very Fine” and is a factor of two smaller than the ACI 224R limits for reinforced concrete members exposed to seawater and deicing chemicals. Again, a 5 s exposure time was employed and 10 images were captured and averaged as described previously. The crack was not detectable using the Canny edge detection algorithm. The crack was visible using the preceding steps in the imaging processing algorithm: (1) averaging of ten images to minimize random noise; (2) Use of the OpenCV THRESH_TRUNC thresholding function to remove the effect of dead pixels; and (3) the use of OpenCV’s Gaussian Blur function to smooth the image and enhance crack visibility. It is noteworthy that the crack was below the SWIR camera’s resolution (0.1 mm per pixel). Nevertheless, the resulting image shown in Fig. 6 demonstrates the ability to identify even small cracks prior to their reaching a size that can threaten a structure’s integrity. The limited visibility of the crack was due to the relatively low resolution of the employed SWIR camera. It can be extrapolated that with a higher resolution camera (e.g., 1 MP camera), one could detect cracks on the order of 0.1 mm at distances on the order of meters.

It is important to note that the Canny algorithm helps to make cracks standout, but the crack shown in Fig. 6 lacked the necessary contrast for Canny to detect it. This was because the crack was below the pixel resolution of the camera, thus pixels contained a mix of the crack and surrounding fluorescing material. The cracks were nevertheless present and can be detected by the human eye. It is also worth noting that though the contrast between the shaded interiors and the fluorescing sample surface makes the cracks visible, it precludes the ability to measure a crack’s depth. That is, the shading of the interiors prevents one from observing or measuring the interiors of cracks.

As described above, the detection of cracks using Near-IR fluorescence imaging was conducted in the presence of indoor laboratory lighting (using a white LED light source); specifically, the sample did not need to be kept in a dark enclosure. A spectroscopic analysis of the light emitted from fluorescent light sources shows that these sources emit no light in the vicinity of the 1140 nm concrete fluorescence^31^. As a result, the demonstrated technique is expected to be effective under fluorescent lighting. Natural daylight, however, does present a complication. The solar spectrum extends into the infrared and includes light emitted at the fluorescence wavelength (1140 nm) which could overwhelm the fluorescence signal. However, there is an H_2_O absorption band centered at 1140 nm. Absorption due to atmospheric water vapor leads to a strong depletion of solar radiance at this wavelength^32, 33^. The question is, whether there is still enough solar radiation at 1140 nm when sunlight reaches the ground to overwhelm light emitted by concrete fluorescence. Depending on the solar angle and atmospheric humidity, direct sunlight may well include too much 1140 nm to use this technique. Diffuse sunlight on cloudy days, though, shows a very strong reduction in 1140 nm light^32, 34^; additional daylight studies would be necessary to determine whether this reduction is to the point where ambient 1140 nm light does not compete with the concrete fluorescence. Indeed, it may be possible to shade the measurement area using a filter-tent made of a Near-IR shielding textile^35^ which filters out the deleterious 1140 nm wavelength from sunlight. In any case, the technique can be employed before dawn and after sunset using appropriate artificial lighting.

## Future work

The work presented in this paper represents a proof of concept demonstration of using near-IR fluorescence to detect cracks in concrete. We have identified additional work that would build upon this demonstration to make it practical crack detection technique. For example:

- The demonstration maximized crack detectability by using LED bars that were parallel to the direction of the cracks which helped optimize the shaded regions within cracks. A practical extension to detect randomly oriented cracks would involve recording several fluorescence images for each sample area, where the LED light was rotated for each image; this would ensure that randomly oriented cracks would have shaded interiors in at least one of the images.
- The demonstration used Gaussian Blurring and the Canny Edge Detection algorithm to address issues such as shadows due to a sample’s surface roughness. Additional studies with different types, grades, surface roughness, and ages of concrete may provide insight for optimizing the post-processing parameters and determining the effectiveness of the technique.
- The proposed technique may be combined with AI routines, which have already shown some success in the detection of cracks in concrete^21^.

## Conclusions

Cracks in concrete samples were detected by recording images of the 1140 nm fluorescence of concrete when excited using red LED light (*λ*_peak_ = 632 nm) while in ambient laboratory lighting. Excitation light was incident on samples at a shallow angular range (θ_*i*_ was between 10 and 40 degrees). The shallow angle was used to ensure that the interior surfaces of cracks received no excitation light. As a result, images show a fluorescing concrete surface with cracks appearing as dark features, due to their lack of fluorescence. Image processing using Canny edge detection from the OpenCV library was used to highlight cracks recorded in the images. Images of artificial cracks having widths 0.2 mm, 0.5 mm, 0.7 mm, 1.2 mm, and 1.5 mm were recorded using a SWIR camera demonstrated the capability to identify cracks approaching the threshold at which damage would occur to concrete structures. A second sample crack having a width of 0.08 mm was also imaged and demonstrated the ability to identify cracks that could be precursors to damage of concrete structures. The primary limitation to the sizes of the cracks detected in the reported work, as well as the distance between the camera and the sample, was the relatively low resolution of the SWIR cameras. The use of a higher resolution camera should enable the detection of cracks from larger distances, on the order of meters, potentially enabling the detection of cracks in bridges, walls, ceilings, and other difficult-to-reach surfaces using mobile non-contact cameras. The proposed method can be combined with unmanned aerial vehicles for future bridge or road crack detection.

## Data Availability

The datasets used and/or analyzed during the current study are available from the corresponding author on reasonable request.
